# Dynamic compliance of the CSF system in iNPH (Part I) - in vitro investigation of the impact of spinal canal stenoses

**DOI:** 10.1186/s12987-026-00805-6

**Published:** 2026-05-09

**Authors:** Anne E. Benninghaus, Kevin Ebers, Chuh-Hyoun Na, Hans Clusmann, Uwe Kehler, Luca Papavero, Klaus Radermacher

**Affiliations:** 1 Chair of Medical Engineering, RWTH Aachen University, Pauwelsstr. 20, 52074 Aachen, Germany; 2 Department of Neurosurgery, University Hospital RWTH Aachen, 52074 Aachen, Germany; 3 Department of Neurosurgery, Asklepios Klinik Altona, 22763 Hamburg, Germany; 4 (formerly) Clinic for Spine Surgery, Schoen Clinic Hamburg Eilbek, 22081 Hamburg, Germany

**Keywords:** Spinal canal stenosis, Compliance, CSF dynamics, iNPH

## Abstract

Spinal canal stenosis is an age-related degenerative condition observed mainly in the cervical and lumbar regions. Recent research has suggested that such stenoses influence cerebrospinal fluid (CSF) dynamics in idiopathic normal pressure hydrocephalus (iNPH) patients by increasing spinal canal flow resistance thereby directly impacting craniospinal dynamic compliance and intracranial pressure (ICP). This study experimentally investigates the effects of cervical and lumbar spinal stenoses on CSF dynamics using a validated in vitro model. To simulate varying degrees of stenosis, controlled reductions in the spinal canal cross-sectional area were applied, and resulting changes in bidirectional cervical CSF flow, ICP, and compliance were measured. The results indicate that mild to moderate stenoses have minimal impact on CSF dynamics, whereas severe stenoses (cervical *<* 33% and lumbar *<* 17% of initial cross sectional area) significantly alter CSF dynamics. These alterations were characterised by reduced dynamic compliance, decreased spinal CSF flow and increased ICP amplitudes up to 7.85 mmHg. These findings suggest that spinal stenoses critically alter key CSF dynamics, potentially contributing to iNPH. Further studies are required, especially on other influencing factors such as age-related changes in viscoelastic properties of the dural sac and the relevance of dynamic compliance of the CSF system, which will be addressed in Part II of this series.

## Introduction

Spinal stenosis is an age-related degenerative condition, most frequently occurring in the cervical [1] and lumbar [2] regions of the spinal canal. It can be caused by a protrusion of the intervertebral disks, a thickening of the ligamentum flavum, the formation of osteophytes, or a combination of these factors [3, 4]. By narrowing the spinal canal, these structural changes may affect cerebrospinal fluid (CSF) dynamics, particularly in aging populations. Another condition associated with aging, idiopathic normal pressure hydrocephalus (iNPH) also correlates with the age of the patients and is characterized by a disturbance in CSF dynamics [5]. Although the pathogenesis of iNPH is still mostly unknown. Some studies have suggested a potential link between iNPH and stenosis. For example, Tominaga et al. found spinal canal stenoses in over 32% of iNPH patients [6, 7]. While existing studies suggest changes in pressure due to stenosis [8], they do not explore the broader impact of quantitative spinal stenoses on overall CSF dynamics (flow and pressure) and especially dynamic compliance in the context of iNPH.

**Fig. 1:**
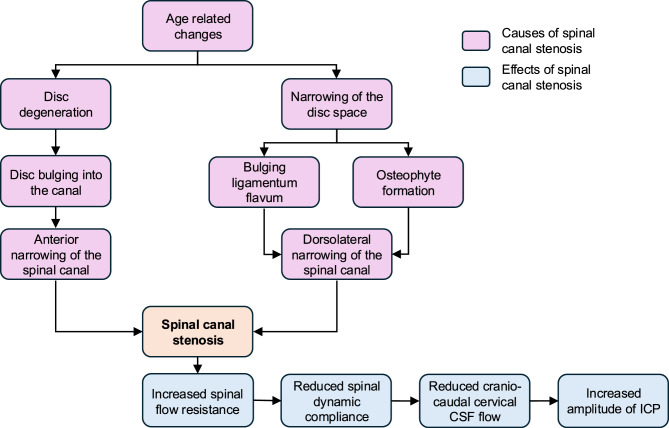
Causes of spinal canal stenosis and possible effects on CSF dynamics

It is reasonable to suggest that age-related alterations in the spine contribute to cervical and lumbar spinal canal stenosis, leading to heightened resistance within the spinal canal and consequently affecting the CSF dynamics. This augmented flow resistance may diminish spinal dynamic compliance, resulting in a decrease in cranio-caudal CSF flow and an increase in intracranial pressure (ICP) amplitudes, which have already been documented in iNPH [9–13]. Verifying this hypothesis (Fig. 1) is important as it would clarify the underlying mechanisms involved and inform therapeutic strategies for the clinical management of iNPH. Therefore, this study aims to address this knowledge gap by experimentally investigating these interactions using a validated in vitro model [14, 15].

## Materials and methods

An in vitro model of the CSF system was developed and validated to analyze its fluid dynamics [14]. The model consists of a ventricular system inside a silicon parenchyma which is connected via the aqueduct to the cranial and spinal subarachnoid space. A cranial and a spinal compliance chamber are connected to cushion the pulsatile CSF flow. To model elastic properties related to static compliance, these chambers are filled with water and air, with an initial air volume of 8 mL in the cranial chamber and 22 mL in the spinal chamber. At baseline, this configuration results in a static compliance of 0.291 mL/mmHg for the cranial chamber and 0.804 mL/mmHg for the spinal chamber. It is assumed that the lumbar end of the spinal canal exhibits the highest compliance value due to the absence of bony boundaries [Tain et al. 2011], which is why the spinal compliance chamber is connected at the lumbar end. Arterio-venous blood flow is controlled by a cam plate-driven pump, which is connected to the cranial compartment and has a stroke volume of 1.09 mL, employing the same cam disk as utilized in the validated model [15]. The CSF dynamics is monitored by an ICP sensor (NEUROVENT, Raumedic, Helmbrechts, Germany) and a spinal ultrasound flow meter (Sonoflow CO.55/060, Sonotec, Halle, Germany). In addition, all sensors were connected to the NI cDAQ-9174 computer data logging system with the module NI 9237 for the pressure sensor and the module NI 9230 for the ultrasound flow sensor, which allowed the signal output to be recorded simultaneously and analyzed with the corresponding manufacturer software DIAdem (National Instruments, Austin, Texas, USA). In this setup, the ICP sensor measures significant changes with an accuracy of 0.034 mmHg and the ultrasound flow meter with 5.9 mL/min. All results were measured over 16 cardiac cycles at 70 beats/min [15].

**Fig. 2:**
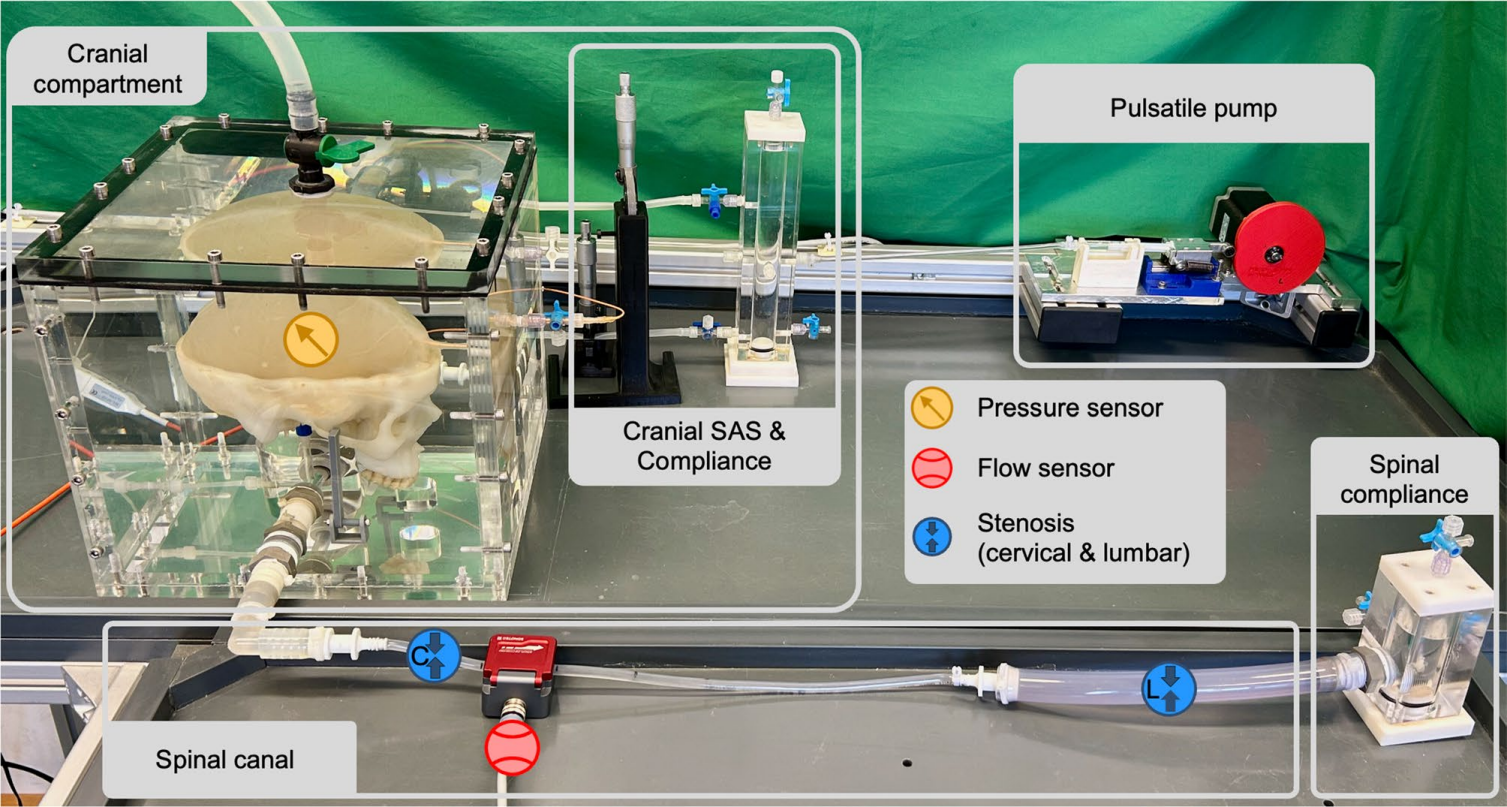
In vitro model of the CSF dynamics including sensors and stenosis positioning

The setup, acquisition and stenosis positions are shown in Fig. 2.

### Physiological in-vitro model of the spinal subarachnoid space

The spinal canal is represented by tubes of various diameters. Although the spinal subarachnoid space is more like a ring-shaped gap with variable flow resistance, it can be approximated as a circular cross section [16]. The inner hydraulic diameter of the spinal canal varies physiologically between 5 and 16 mm. Consequently, in the model, the spinal canal is represented by tubes with inner diameters of 5 and 16 mm, corresponding to a total characteristic length of 70 cm, which reflects the average anatomical length of the spinal canal in adult males and is relevant for hydrostatics [16, 17]. Inclusion of the spinal cord or spinal nerve roots is not required in this model, as their influence or movement are not the subject of this work. The primary concern is to ensure physiological resistance due to the hydraulic diameter.

### Compliance calculation

In general, compliance (C) refers to a system’s or tissue’s ability to accommodate changes in volume (∆V), with the magnitude of compliance being determined by the corresponding change in pressure (∆p). The mathematical relationship is described by Marmarou et al. as follows [18]:

1 $$C = {{\Delta V} \over {\Delta p}}$$

The determination of the system boundaries and associated pressure and volume changes of the cranial and spinal compartments is crucial for the definition of cranial and spinal compliance. To validate compliance in vivo, it must be described by clinically measurable pressures and flows. The compliance of the cerebrospinal fluid (CSF) system is commonly assessed using an infusion test. In this procedure, a constant volume flow, a flow maintained at constant pressure, or a bolus infusion is administered via lumbar puncture, and the resulting pressure response is measured [19, 20]. It is important to note that the infusion test relies on the artificial addition of volume to the system, which does not reflect the physiological dynamic response to changes in CSF volume over a cardiac cycle. Particularly when volume is introduced slowly into the CSF system, it does not capture a dynamic response; instead, it reflects static compliance. Static compliance can be understood as the elastic response of the system and can be effectively modeled using either a spring or an air volume [14].

An alternative method for calculating dynamic compliance over one cardiac cycle is presented by Lokossou et al. [21]:

2 $$\begin{aligned} C_ = \frac{\Delta IVC}{\Delta ICP}\label{eq:compliance-rest}\end{aligned}$$

The volume changes of blood and CSF are combined to assess the intracranial volume change (∆IVC) over one cardiac cycle. This value is then divided by the amplitude of intracranial pressure (∆ICP) to derive the intracranial compliance, expressed in mL/mmHg. As a magnitude reference for this methodology, Lokossou et al. report intracranial compliance values of 0.23 *±* 0.15 mL/mmHg [21]. This approach was selected because it allows for a dynamic calculation of compliance using an established method, facilitating comparability with values reported in the literature.

### Spinal canal stenoses

Spinal canal stenoses can be divided into two categories, cervical and lumbar, since thoracic stenoses are rare [22, 23]. Due to the different diameters and incoming flow velocities in the cervical and lumbar regions, it is reasonable to examine stenoses at both levels despite there being only one spinal compliance chamber at the lumbar end. In order to model spinal canal stenosis on the test bench, the cross-sectional area of the spinal canal should be reduced, as it is largely responsible for flow resistance. In the case of a squeezed tube, which no longer has a circular cross section, the area cannot be calculated using the radius alone. The cross-sectional area changes, but the circumference remains constant.

The area *A*_2_ is divided into a circle and a rectangle (Table 1, Eq. 4):

3 $$\begin{aligned} A_1 &= \frac{\pi \cdot d_1^2}{4} \label{eq:area_relaxed}\\ A_2 &= \frac{\pi \cdot d_2^2}{4} + d_2 \cdot l \label{eq:area_squeezed}\end{aligned}$$

4 $$\begin{aligned} A_1 &= \frac{\pi \cdot d_1^2}{4} \label{eq:area_relaxed}\\ A_2 &= \frac{\pi \cdot d_2^2}{4} + d_2 \cdot l \label{eq:area_squeezed}\end{aligned}$$

**Table 1:** Calculating the area (A) and circumference (C) of a relaxed (1) and a squeezed tube (2)

	(1) Relaxed tube	(2) Squeezed tube
		
$A$	$\frac{\pi \cdot d_1^2}{4}$	$\frac{\pi \cdot d_2^2}{4} + d_2 \cdot l$
$C$	$\pi \cdot d_1$	$\pi \cdot d_2 + 2 \cdot l$

The circumference *C*_2_ is given by:

5 $$\begin{aligned} C_1 &= \pi \cdot d_1 \label{eq:circumference_relaxed}\\\end{aligned}$$

6 $$\begin{aligned} C_2 &= \pi \cdot d_2 + 2 \cdot l \label{eq:circumference_squeezed}\end{aligned}$$

Since the circumference of the tube does not change when it is squeezed, the following applies:

7 $$\begin{aligned} C_2 = C_1 = \pi \cdot d_1 \label{eq:constant_circumference}\end{aligned}$$

Substituting *C*_2_ from Eq. 7, in Eq. 6 and solved for *l*

8 $$\begin{aligned} l = \frac{\pi \cdot d_1 - \pi \cdot d_2}{2} = \frac{C_1 - \pi \cdot d_2}{2} \label{eq:length}\end{aligned}$$

Substituting the Eq. 8 into the Eq. 4 gives the following result:

9 $$\begin{aligned} A_2 = \frac{\pi \cdot d_2^2}{4} + d_2 \cdot \frac{C_1}{2} - \frac{\pi \cdot d_2^2}{2} \label{eq:final_area}\end{aligned}$$

Our choice of a linear reduction in area was guided by the findings of Mortensen et al. [Mortensen et al. 2005], which highlighted the significance of both area and circumference in influencing resistance. A micrometer screw, adjustable to an accuracy of 10 µm, was used to compress the tube at both stenosis locations. By applying external compression to both tubes, a smooth, gradual narrowing is achieved rather than an abrupt constriction. This geometry reduces turbulence and functions analogously to a Venturi tube, though the cross-section at the point of constriction is non-circular [24].

#### Cervical stenosis

**Fig. 3:**
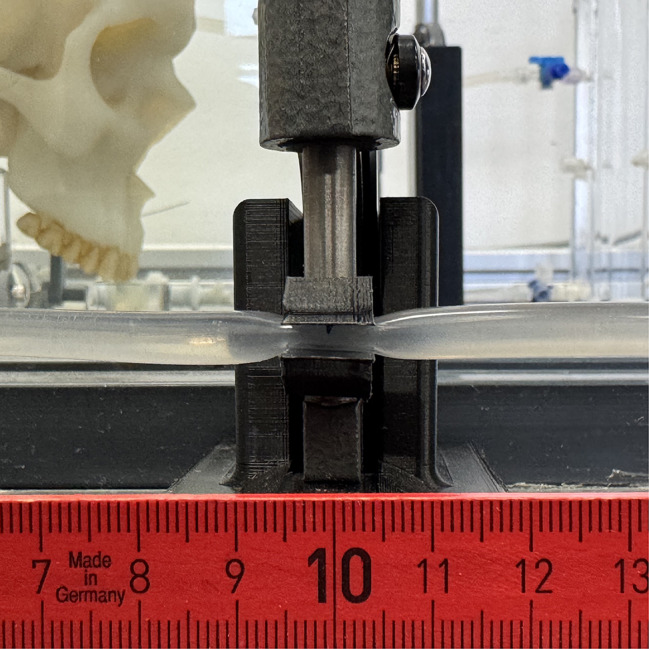
Cervical tube compression with 10 mm length along the tube using a micrometer screw for varying diameters

For the investigation of cervical stenoses, the circular cross section of the tube is reduced linearly for each measurement until complete closure using a micrometer screw (Fig. 3). The cervical tube has an inner diameter of 5 mm and a wall thickness of 1 mm. The initial area of the relaxed tube (*A*1) can be calculated from the inner diameter according to Table 2 and is 19.635 mm^2^. The area of the squeezed tube (*A*2) can be calculated according to Table 2. In total, the cross-sectional area should be reduced to 0 mm^2^ in seven measurements. This results in the following measurements (Table 2).

**Table 2:** Calculation of the diameters for the cervical stenosis based on the area

Measurement	Area (%)	Area (mm^2^)	Inner Diameter (mm)
1	100.00	19.635	5.00
2	83.33	16.362	2.96
3	66.67	13.090	2.11
4	50.00	9.817	1.46
5	33.33	6.545	0.92
6	16.67	3.272	0.44
7	0.00	0.000	0.00

#### Lumbar stenosis

**Fig. 4:**
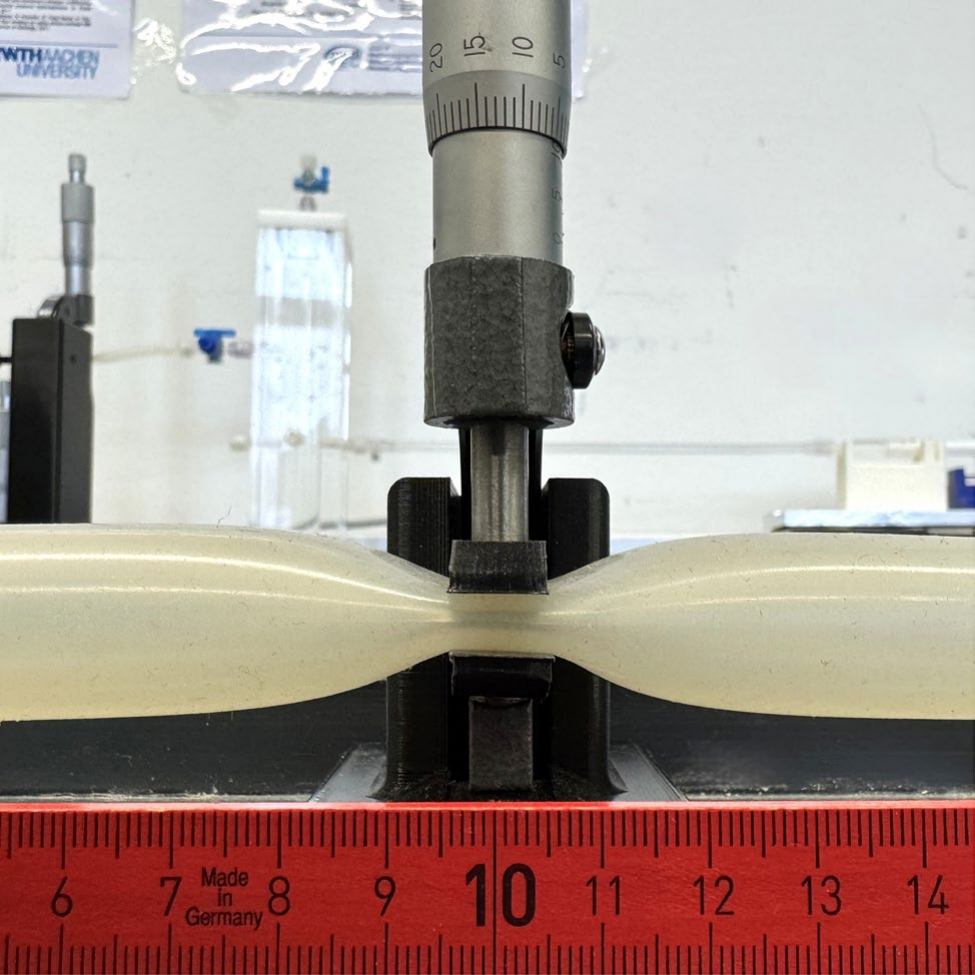
Lumbar tube compression with 10 mm length along the tube using a micrometer screw for varying diameters

**Fig. 5:**
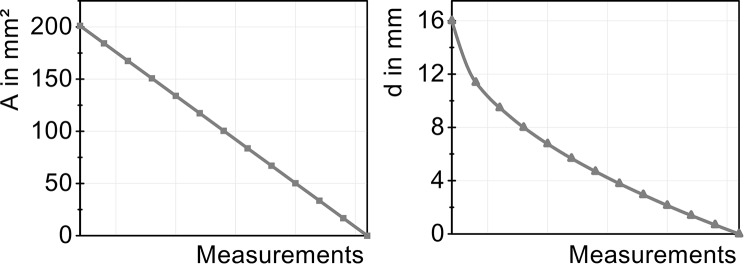
Illustration of the varying cross-sectional area *A* (left) and the resulting inner diameter *d* (right) for the 13 measurements

Based on the measurements of the cervical stenoses, a similar procedure was used for the lumbar region. The cross-sectional area was reduced from 100% to 0% divided into 13 measurements, as shown in Table 3. Figure 4 illustrates the setup for lumbar stenosis in the model, while Fig. 5 shows the cross-sectional area of the tube, which decreases linearly with each measurement in relation to the corresponding inner diameter.

**Table 3:** Calculation of the diameters for the lumbar stenosis based on the area

Measurement	Area (%)	Area (mm^2^)	Inner Diameter (mm)
1	100.00	201.062	16.00
2	91.67	184.307	11.38
3	83.33	167.552	9.47
4	75.00	150.796	8.00
5	66.67	134.041	6.76
6	58.33	117.286	5.67
7	50.00	100.531	4.69
8	41.67	83.776	3.78
9	33.33	67.021	2.94
10	25.00	50.265	2.14
11	16.67	33.510	1.39
12	8.33	16.755	0.68
13	0.00	0.000	0.00

## Results

### Cervical stenosis

**Fig. 6:**
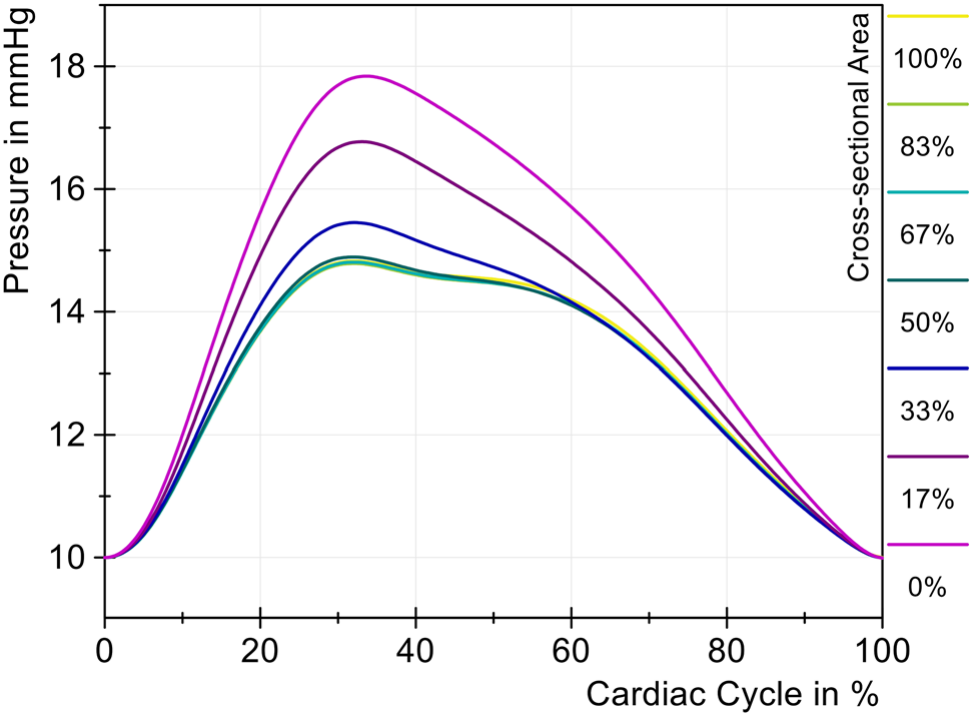
Modification of the ICP with different cross-sectional areas (cervical stenosis)

For the simulation of cervical stenosis, the cervical tube section was compressed. The remaining open cross-sectional area was given as a percentage below and was 19.635 mm^2^ at 100%. Further values can be taken from Table 2. Figure 6 showes the pressure change of the ICP associated with different cross-sectional areas over one cardiac cycle. The open tube (100%, yellow) was compressed to cause a linear decrease in the cross-sectional area with each measurement until a complete stenosis was reached, and thus a complete closure of the tube (0%, purple) was achieved.

**Fig. 7:**
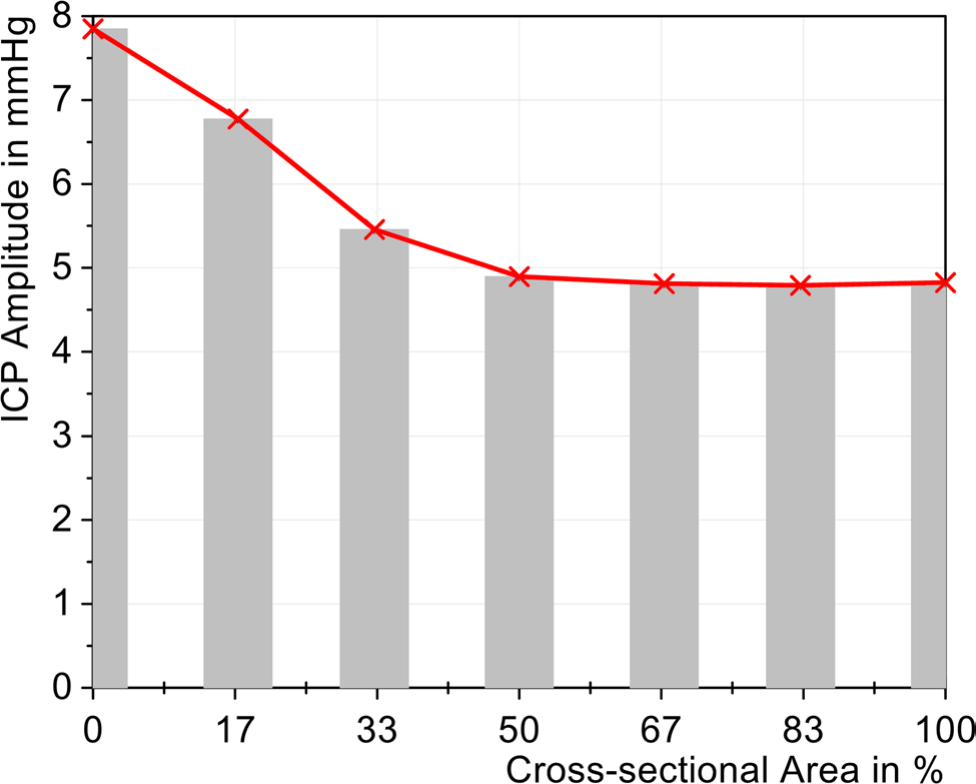
Change of ICP amplitude with different cross-sectional areas (cervical stenosis)

It can be seen that the ICP curve hardly changed with a reduction of up to 50% in area. Although the ICP amplitude increased by 0.07 mmHg and thus already represented a significantly measurable change, this increase of 1.5% can be classified as rather small. Only a significant increase in the ICP amplitude was observed starting with a reduction in the area to 33%, which exceeded the limit value of 5 mmHg, as can be seen in Fig. 7. The amplitude then continued to increase as the reduction progresses and reached a maximum amplitude of 7.85 mmHg when the tube was completely blocked. The rate of pressure rise and fall also changed significantly at an open area of 33%, indicating reduced dynamic compliance.

**Fig. 8:**
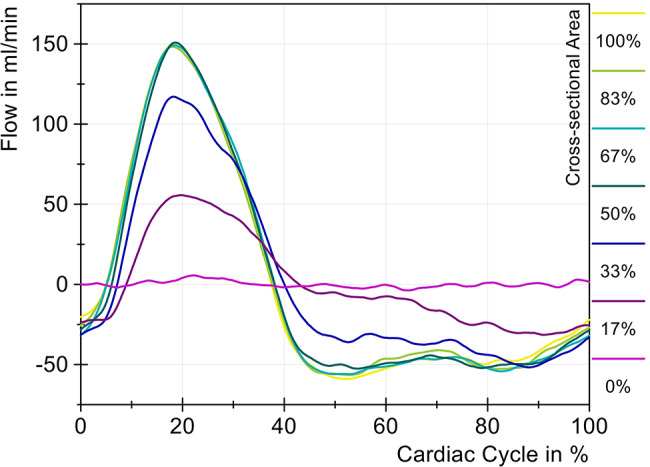
Change in cervical flow with different sized cross-sectional areas (cervical stenosis)

With decreasing area, only one pressure peak was observed, although the time of the maximum shifted only slightly. Overall, it can be concluded that the changes in the cervical cross-sectional area on the pressure are only significant starting with a reduction to 33% of the initial area and then increase significantly with further reduction. Similar to the pressure changes, a noticeable change in flow measurements, shown in Fig. 8, was only observed from 33% onward. The difference in maximum cranio-caudal flow between 100% and 50% cross-sectional area was only 0.2 mL/min and thus not statistically significant. However, starting at 33%, the maximum cranio-caudal flow decreased from 150.8 mL/min to 117.1 mL/min, corresponding to a reduction of 22.3%. In a relative cross-sectional area of 17%, a loss of 63.1% was measured in the maximum cranio-caudal flow. When the tube was fully closed, no flow was detected, as expected. The constriction was located directly cranial to the sensor, preventing further flow generation. The slight fluctuations in the purple curve (0%) were due to artifacts in the ultrasound sensor.

It can be seen that the negative flow in the caudo-cranial direction was less affected than in the opposite direction. The reduction to 33% led to a decrease in caudo-cranial flow amplitude by 12.2% and was significantly lower than before in caudal direction.

**Fig. 9:**
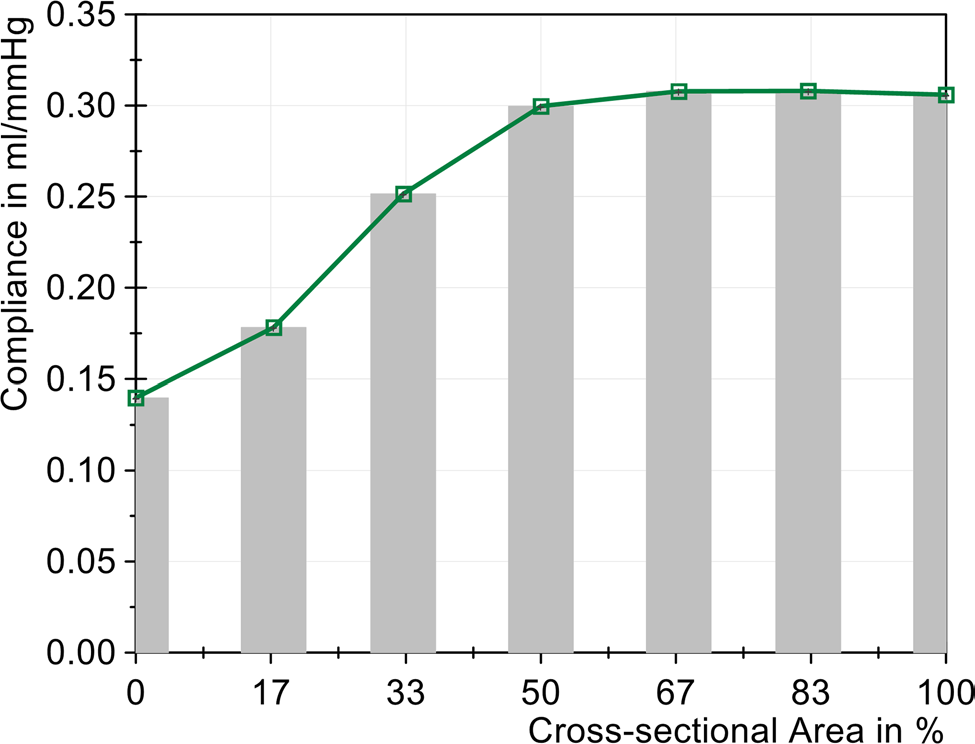
Change in compliance with different sized cross-sectional areas (cervical stenosis)

The compliance calculation (Fig. 9) shows a substantial change in the compliance values below a reduction to 33% of the cross-sectional area of the tube, which was reduced by 54.3% when the area was reduced from 100% to 0%. This observation aligns with the ICP amplitude, suggesting a plateau in the compliance calculations between 100% and 50%.

### Lumbar stenosis

Similar to cervical stenosis, the principle of tube compression was also applied in the lumbar region. At the start of the measurements, the tube had a maximum diameter of 16 mm and a cross-sectional area of 201.062 mm^2^, which was gradually reduced.

**Fig. 10:**
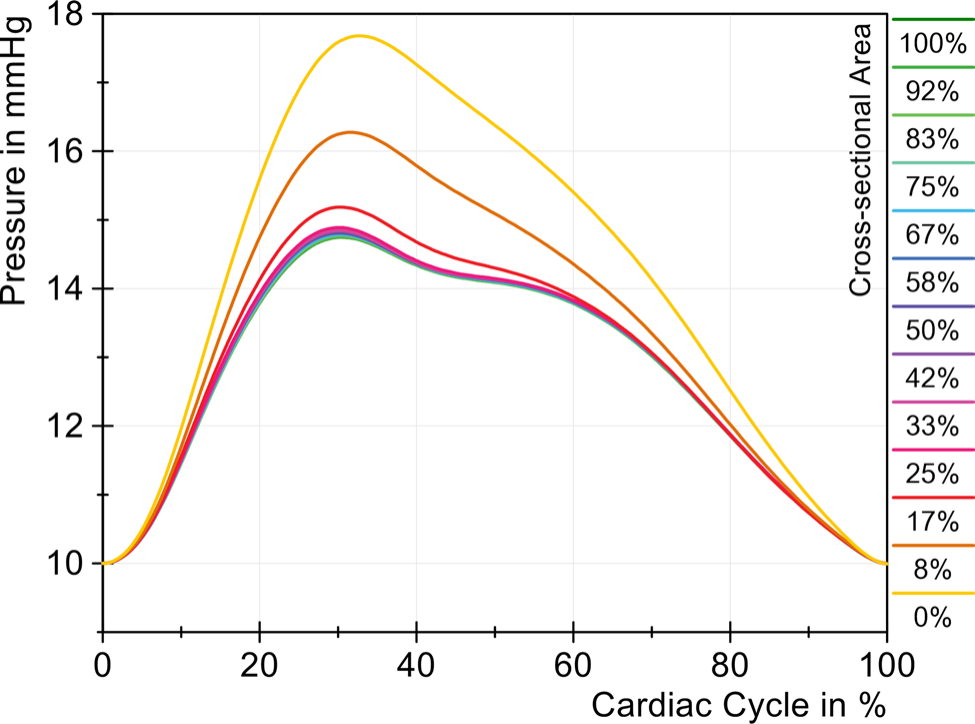
Pressure changes with different cross-sectional areas (lumbar stenosis)

Figure 10 illustrates the change in ICP with decreasing cross-sectional area over one cardiac cycle. The initial area (100%, green) was gradually reduced until the tube was fully compressed (0%, yellow). The ICP curve remained constant until the cross-sectional area was reduced to 17% (red). Both the positive and negative rates of pressure increase were more prominent in severe stenosis (*<* 17%), mainly due to the increasing pressure amplitudes. The timing of the pressure maxima shifted only slightly, by 2.2% of the cardiac cycle, as the cross-sectional area decreased from 100% to 0%. Additionally, the two pressure peaks merged.

**Fig. 11:**
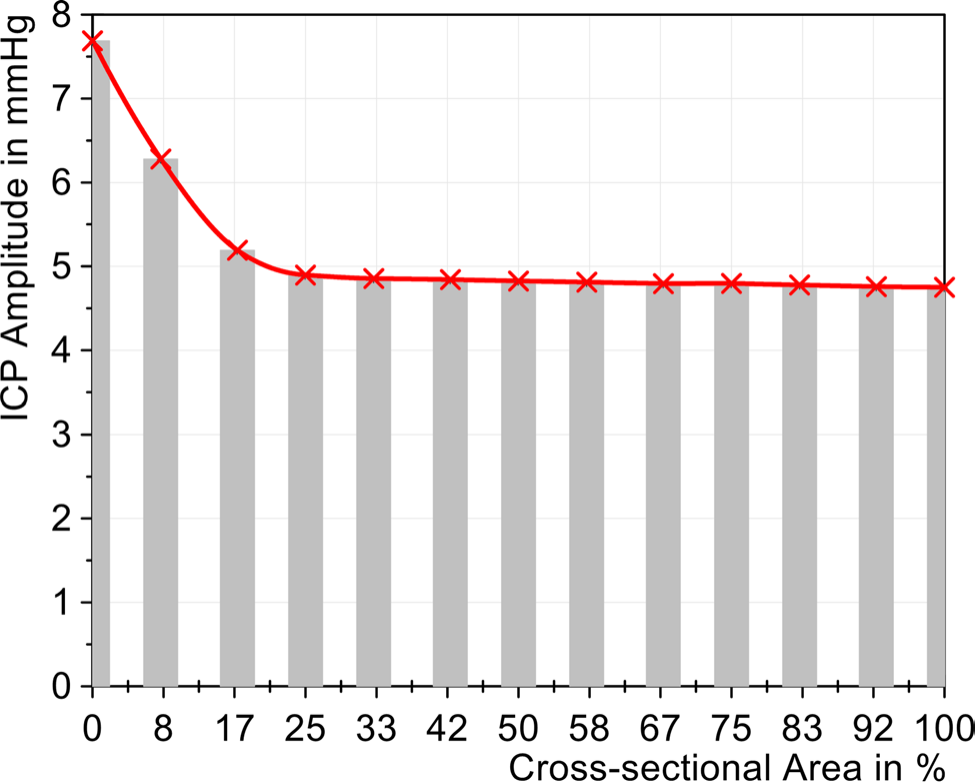
Change in ICP amplitude with different cross-sectional areas (lumbar stenosis)

The ICP amplitude increased almost linearly up to a 25% reduction in relative area, rising from 4.75 mmHg to 4.90 mmHg, as shown in Fig. 11. Upon reduction of the area to 17%, the ICP amplitude increased to 5.19 mmHg. With complete lumbar stenosis (0%), the resulting amplitude reached 7.69 mmHg, representing an increase of 61.9%.

**Fig. 12:**
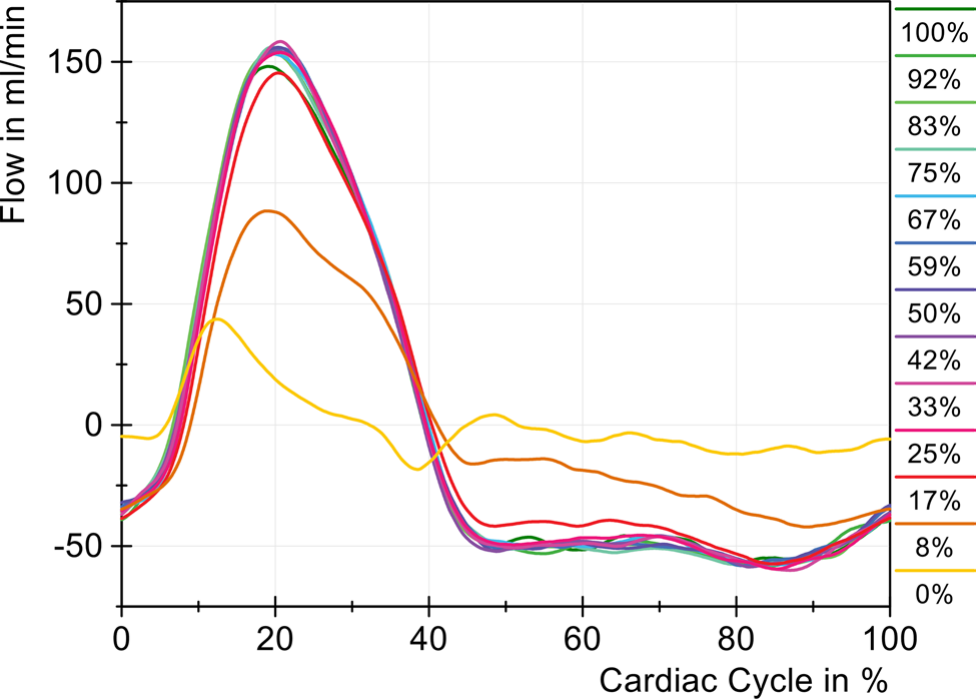
Change in cervical CSF flow with different sized cross-sectional areas (lumbar stenosis)

The results of the flow measurements (Fig. 12) for lumbar stenosis are comparable to those observed in cervical stenosis. A reduction to 25% of the initial area did not lead to significant changes in flow. Only at 17% (red) does a slight decrease in bidirectional flow amplitudes became visible, which became more distinct at 8% (orange) and 0% (yellow). The remaining amplitude of 43.7 mL/min in the cranio-caudal direction and *−*18.4 mL/min in the caudocranial direction under complete compression was attributed to the compliance of the lumbar silicone tube. Consequently, part of the spinal volume could still pulsate up to the stenosis, which resulted in a small but measurable cervical flow with a stroke volume of only 0.074 mL compared to 0.428 mL at baseline (100% area).

**Fig. 13:**
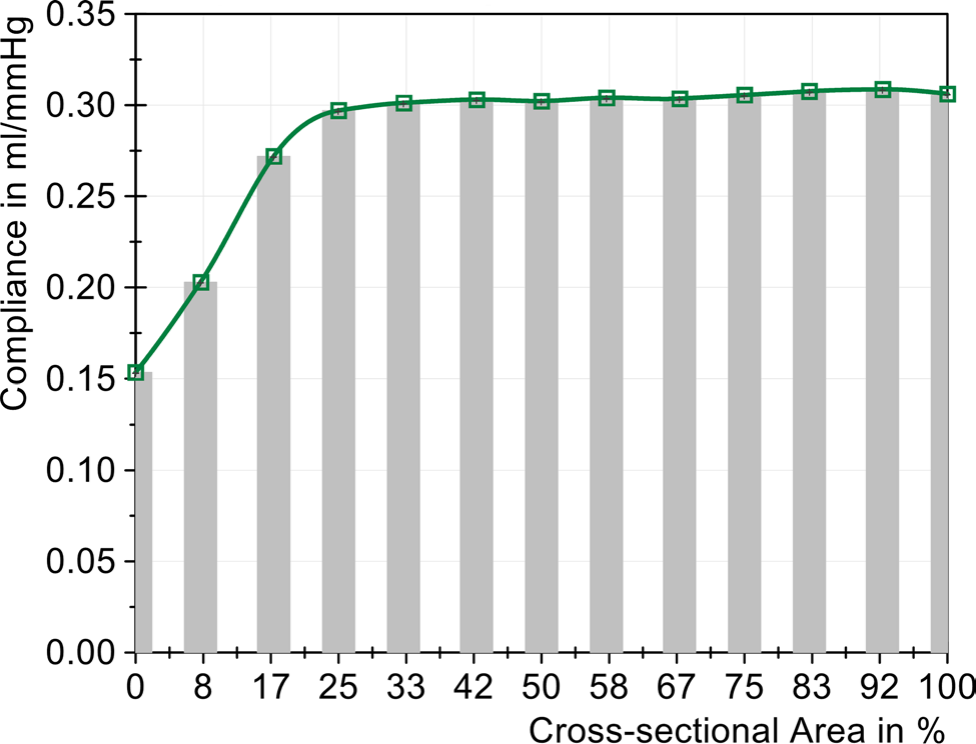
Change in compliance with different sized cross-sectional areas (lumbar stenosis)

The compliance change, mapped in Fig. 13, presented a similar picture to that of the cervical stenosis in Fig. 9. The compliance decreased slightly in a linear way from 100% to 25%. A substantial decline in compliance was only observed with a reduction to 17% cross-sectional area. With complete compression, the compliance was reduced by approximately half, from 0.306 mL/mmHg to 0.151 mL/mmHg. The change in compliance, despite a constant air volume, showed the significant influence of severe stenoses on the CSF system.

## Discussion and outlook

The objective of this study was to experimentally evaluate the hypothesis that reduced dynamic compliance due to stenosis results in decreased cervical cranio-caudal CSF flow and heightened ICP amplitudes (Fig. 1). To test this hypothesis, we modeled both cervical and lumbar spinal canal stenoses and measured the resulting CSF dynamics. The findings of this study support this hypothesis, highlighting the intricate relationship between the morphology of the spinal canal and the flow dynamics of the CSF. Specifically, we found that mild to moderate stenoses had little or no effect on the craniospinal fluid system, indicating that small reductions in flow resistances may be compensated for. In contrast, severe stenosis, in which the changes were significant, corresponded to cervical constriction of up to 33% (6.545 mm^2^) and lumbar of up to 17% (33.510 mm^2^) of the initial cross-sectional area. This non-linear behaviour is consistent characteristics of Venturi-type geometries, in which the discharge coefficient, which resembles energy loss, exhibits a non-linear dependence on the diameter ratio [24], qualitatively supporting our experimental observation that compliance is measurably affected only when the tubes are severely occluded. A specific scenario arose when cervical or lumbar stenosis resulted in complete occlusion of the spinal canal. In such instances, access to the air chamber responsible for spinal compliance was blocked. Consequently, in cases of complete occlusion, static compliance was restricted solely to the cranial compartment, and ICP amplitudes of 7.85 mmHg were recorded, exceeding the limit value of 5 mmHg defined by Eide et al. [25–27].

Although the air volume in both chambers was kept constant throughout the entire test, a non-linear reduction in dynamic compliance was measured with increasing tube compression and, consequently, stenosis severity. These results underscore the necessity of distinguishing between different types of compliance. In our model, we maintained static compliance, which corresponds to the elastic behavior within the compliance chambers. Nonetheless, the compliance values change according to Lokossou’s calculations because they incorporate dynamic aspects over a cardiac cycle. Therefore, a static characterization of compliance [Benninghaus 2019] cannot be directly compared to the dynamic calculation approach. All calculated values of the dynamic compliance in our model (0.140 *−* 0.309 mL/mmHg) align well with the values reported by Lokossou et al. (0.23 *±* 0.15 mL/mmHg) [21], reinforcing the relevance of our model and suggesting that it reflects physiological conditions. Our results suggest that severe spinal stenoses strongly affect overall CSF dynamics and dynamic compliance, which is considered an essential parameter with respect to iNPH [28–31]. However, this correlation has not yet been described in the literature. Future research should aim to correlate our defined severe stenosis thresholds (33% for cervical and 17% for lumbar of the cross-sectional area) with those commonly observed in patients with idiopathic normal pressure hydrocephalus (iNPH). Although we currently lack specific population data, this area warrants clinical investigation to better understand the impact of these stenosis degrees on patient outcomes.

Spinal imaging is not the gold standard for the diagnosis of iNPH in most countries in Europe and North America, since ventriculoperitoneal shunts are the most common [32]. In contrast, in Japan spinal imaging is used due to the application of lumboperitoneal shunts [33], and initial studies on the incidence of patients with stenosis and iNPH have been conducted [6, 7]. These studies show that iNPH patients with lumbar stenoses show only a slight improvement in gait despite shunt surgery compared to iNPH patients without stenosis [6, 7]. Consequently, persistent postoperative gait disturbance in patients with NPH could be caused by concomitant spinal stenosis. Against this background, spinal imaging generally appears to be recommended as part of a differential diagnosis in the context of iNPH. Additional studies on the influence of different morphological changes in the spinal canal related to iNPH, especially in correlation with stenosis, would be desirable and necessary to verify model-based results.

Despite the findings of this study, limitations must be acknowledged. Simplifications were made, including the geometry of the spinal canal as a circular cross section and the representation of a purely static compliance and no viscoelastic behavior within the CSF system. Potential long-term effects of spinal canal stenosis on CSF dynamics, as well as its influence on iNPH, require further investigation. While this study highlights the significant impact of stenosis on CSF dynamics, other age-related factors, such as changes in the viscoelasticity properties of tissue forming the boundaries of the spinal compartment, also play a crucial role in dynamic compliance and associated alterations in CSF dynamics. These analyses have been the objective of our further studies and will be outlined in an upcoming publication (Part II).

## Data Availability

No datasets were generated or analysed during the current study.
